# The relative brain signal variability increases in the behavioral variant of frontotemporal dementia and Alzheimer’s disease but not in schizophrenia

**DOI:** 10.1177/0271678X241262583

**Published:** 2024-06-19

**Authors:** Timo Tuovinen, Jani Häkli, Riikka Rytty, Johanna Krüger, Vesa Korhonen, Matti Järvelä, Heta Helakari, Janne Kananen, Juha Nikkinen, Juha Veijola, Anne M Remes, Vesa Kiviniemi, on behalf of the Frontotemporal Lobar Degeneration Neuroimaging Initiative, Howard Rosen

**Affiliations:** 1 Oulu Functional NeuroImaging, Research Unit of Health Sciences and Technology, University of Oulu, Oulu, Finland; 2 Medical Research Center, Oulu University Hospital, The Wellbeing Services County of North Ostrobothnia, Oulu, Finland; 3 Neurology, Hyvinkää Hospital, The Wellbeing Services County of Central Uusimaa, Hyvinkää, Finland; 4 Research Unit of Clinical Medicine, Neurology, University of Oulu, Oulu, Finland; 5 Neurology, Neurocenter, Oulu University Hospital, The Wellbeing Services County of North Ostrobothnia, Oulu, Finland; 6 Clinical Neurophysiology, Oulu University Hospital, The Wellbeing Services County of North Ostrobothnia, Oulu, Finland; 7 Department of Oncology and Radiotherapy, Oulu University Hospital, The Wellbeing Services County of North Ostrobothnia, Oulu, Finland; 8 Research Unit of Clinical Medicine, Department of Psychiatry, University of Oulu, Oulu, Finland; 9 Department of Psychiatry, Oulu University Hospital, The Wellbeing Services County of North Ostrobothnia, Oulu, Finland; 10 Research Unit of Clinical Medicine, Neurology, University of Oulu, Oulu, Finland; 11 Clinical Neurosciences, University of Helsinki, Helsinki, Finland; 12 Biocenter Oulu, University of Oulu, Oulu, Finland

**Keywords:** Alzheimer’s disease, frontotemporal dementia, schizophrenia, functional MRI, brain signal variability

## Abstract

Overlapping symptoms between Alzheimer’s disease (AD), behavioral variant of frontotemporal dementia (bvFTD), and schizophrenia (SZ) can lead to misdiagnosis and delays in appropriate treatment, especially in cases of early-onset dementia. To determine the potential of brain signal variability as a diagnostic tool, we assessed the coefficient of variation of the BOLD signal (CV_BOLD_) in 234 participants spanning bvFTD (n = 53), AD (n = 17), SZ (n = 23), and controls (n = 141). All underwent functional and structural MRI scans. Data unveiled a notable increase in CV_BOLD_ in bvFTD patients across both datasets (local and international, p < 0.05), revealing an association with clinical scores (CDR and MMSE, r = 0.46 and r = −0.48, p < 0.0001). While SZ and control group demonstrated no significant differences, a comparative analysis between AD and bvFTD patients spotlighted elevated CV_BOLD_ in the frontopolar cortices for the latter (p < 0.05). Furthermore, CV_BOLD_ not only presented excellent diagnostic accuracy for bvFTD (AUC 0.78–0.95) but also showcased longitudinal repeatability. During a one-year follow-up, the CV_BOLD_ levels increased by an average of 35% in the bvFTD group, compared to a 2% increase in the control group (p < 0.05). Our findings suggest that CV_BOLD_ holds promise as a biomarker for bvFTD, offering potential for monitoring disease progression and differentiating bvFTD from AD and SZ.

## Introduction

Alzheimer’s disease (AD) and the behavioral variant of frontotemporal dementia (bvFTD) are the leading causes of early-onset dementia.^1,2^ These are neurodegenerative diseases characterized by clinical, genetic, and pathological heterogeneity.^2–5^ AD mostly manifests as amnestic syndrome, while bvFTD encompasses changes in personality and behavior, such as apathy, disinhibition, hyperorality, executive dysfunction, and compulsive behaviors. However, there are often significant overlapping similarities in the clinical presentation of these diseases and atypical variants have been recognized.^6–12^ Additionally, bvFTD shows significant symptomatic overlap with non-degenerative primary psychiatric disorders, including schizophrenia (SZ).^13–16^ Both SZ and bvFTD can be characterized by a profound alteration in personal and social conduct, and the clinical presentation of bvFTD can deviate from that of typical memory disorders.^14,17,18^

Differentiating among the various neurodegenerative causes of dementia enables affected individuals and their families to receive suitable treatment, support, and care. Despite extensive efforts to establish refined clinical guidelines for differential diagnosis, the diagnostic accuracy remains unsatisfactory.^11,15,16,19–26^ The sensitivity of the criteria for possible bvFTD ranges from 75% to 95%, and for probable bvFTD from 64% to 85%.^10,23,24,26,27^ The specificity of the criteria for possible bvFTD ranges from 27% to 82%, and for probable bvFTD from 85% to 95%.^10,23,24,26,27^ Current practice highlights the importance of imaging in the distinguishing bvFTD from other neurodegenerative disorders. However, visual evaluation of magnetic resonance imaging (MRI) requires the expertise of an experienced neuroradiologist and provides only 59% sensitivity and 80% specificity in distinguishing from other common dementing diseases.^26^ It is also susceptible to inter-rater differences. Delayed diagnosis is common, with half of bvFTD patients initially receiving a psychiatric diagnosis, and the average diagnostic delay being up to five years from symptom onset. Conversely, patients with primary psychiatric disorders are often misdiagnosed with bvFTD.^13,15,16,25^

Protein dyshomeostasis seems to be the common mechanism of these neurodegenerative diseases. Additionally, cerebrovascular dysfunction (CVD) has now been recognized as a potential contributor to the onset and progression of neuronal degeneration, strongly linked to neuroinflammation, neurodegeneration, and cognitive decline.^28–31^ Age, cerebrovascular diseases, and hypertension are known risk factors for AD.^21^ Recent studies have shown that the cardiovascular burden is also higher in sporadic FTD, especially among patients with the bvFTD phenotype.^32^ Even though bvFTD and SZ have overlapping neuropsychological findings and neuropsychiatric symptoms, CVD, cardiovascular diseases, and hypertension are not considered risk factors for SZ.^33,34^

Blood oxygenation level dependent (BOLD) imaging using functional MRI has shown that these aforementioned risk factors are reflected in the variability of cerebrovascular pulsatility.^35–41^ Although a BOLD signal has mainly been used to measure hemodynamic responses to neuronal activity, it also reflects underlying physiological factors such as cerebral blood flow, hemodynamics, respiration, and metabolism.^36,42–52^ Recent studies have emphasized that the BOLD signal is not a static measure, showing intrinsic variability over time and between individuals.^53–57^ Fast BOLD imaging reflects both coupled neurovascular activity as well as hydrodynamic physiological factors such as the glymphatic mechanism: its variability has been increasingly used as an indicator of neurovascular state in clinical investigations.^35–41^ We previously studied brain signal variability using coefficient of variation of the BOLD signal (CV_BOLD_),^38,40,41,58^ where we detected a replicable and progressive increase in brain CV_BOLD_ solely in the AD patients, constituting a robust biomarker for clearly differentiating AD cases from controls.^41^ Based on these earlier reports and the findings on the risk factors mentioned previously, we hypothesized that the CV_BOLD_ signal could also be differentially altered in bvFTD and SZ.

In this study, our objectives were to examine whether CV_BOLD_ is altered in bvFTD and/or SZ, and if it could be used in differential diagnostics. We further investigated whether CV_BOLD_ could be used as a non-invasive MR-based biomarker for distinguishing bvFTD and SZ from imaging setup matched healthy control data. We also verified our results by using two independent bvFTD datasets.

## Methods

### Participants

The study sample included 234 individuals from three independent datasets, including 53 patients with bvFTD, 23 patients with SZ, 17 patients with AD who had been studied previously, and 141 healthy controls. Participant datasets and baseline characteristics are shown in Table 1. Ethical approvals were granted by the relevant research ethics committees across the sites. For dataset 1, the NIFD study was approved by the University of California in San Francisco (UCSF) institutional review board. Our research uses publicly available, previously collected and fully anonymized data, thus no additional ethical approval was required. All research protocols for the local datasets (2 and 3) were approved by The Ethics Committee of the Northern Ostrobothnia Hospital District in Finland (92/2002, 11/2008, 94/2011). Written informed consent was obtained from all participants or their assigned legal guardians. Research was conducted in accordance with the Helsinki declaration.

**Table 1.** Overview of study participants.

Dataset 1 – NIFD	Participants	Age at MRI (years± SD)	Female (%)	Education (years)	MMSE	CDR
bvFTD patients	35	61.8 ± 6.6	14 (40 %)^a^	18	23.2 ± 5.4^a^	1.3 ± 0.6^a^
Controls	92	64.7 ± 9.3	58 (62 %)	21	29.3 ± 0.9	0.0 ± 0
Dataset 2 – local dementia study	Participants	Age at MRI (years± SD)	Female (%)	Education (years)	MMSE	FBImod
bvFTD patients	18	60.2 ± 7.3	9 (50 %)	NC	24.2 ± 4.1^a^	23.5 ± 4.7
AD patients	17	60.0 ± 5.4	11 (65 %)	NC	22.9 ± 2.6^a^	NC
Controls	24	60.0 ± 5.1	12 (50 %)	NC	29.0 ± 1.1	NC
Dataset 3 – NFBC 66	Participants	Age at MRI (years± SD)	Female (%)	Education (years)	PANSS total	SOFAS
SZ patients	23	43.2 ± 0.8	10 (43 %)	NA	65 ± 23.3	49 ± 14.2^a^
Controls	25	43.5 ± 0.8	8 (32 %)	NA	NC	82 ± 13.9

Descriptive demographic characteristics of the groups. Values represent mean ± standard deviation or N (%). [Range]. NC: not collected; NA: not available; MMSE: Mini-Mental State Examination (maximum total score is 30); CDR: Clinical Dementia Rating (maximum total score is 3); FBImod: Modified Frontal Behavioral Inventory (maximum total score is 72); PANSS: Positive and Negative Syndrome Scale; SOFAS: Social and Occupational Functioning Assessment Scale (maximum total score is 100).

a Patients versus controls, where p < 0.05.

All bvFTD participants met the International Behavioral Variant FTD Criteria Consortium (FTDC) revised guidelines 2011 for the diagnosis of bvFTD.^10^ All the patients of the AD group met the NINCDS-ADRDA (National Institute of Neurological and Communicative Disorders and Stroke and the Alzheimer’s Disease and Related Disorders Association) criteria for probable AD.^20^ SZ patients met the diagnostic criteria using a Structured Clinical Interview for DSM-IV (SCID-I).^59^

**Dataset 1** was obtained from the frontotemporal lobar degeneration neuroimaging initiative (FTLDNI also known as NIFD), through the LONI portal (http://ida.loni.usc.edu). FTLDNI was funded through the National Institute of Aging and started in 2010: its primary goals were to identify neuroimaging modalities and methods of analysis for tracking frontotemporal lobar degeneration (FTLD) and to assess the value of imaging versus other biomarkers in diagnostic roles. The Principal Investigator of NIFD was Dr. Howard Rosen, MD at the University of California, San Francisco. FTLDNI is a multicentric longitudinal database, collecting MRIs, PET, and CSF biomarkers in FTD patients and age-matched controls. All patients were clinically diagnosed by a multidisciplinary consensus panel.^60^ The data are the result of collaborative efforts at three sites in North America. For up-to-date information on participation and protocol, please visit http://memory.ucsf.edu/research/studies/nifd. The dataset included 35 patients with bvFTD and 92 elderly controls. This dataset also contained follow up data consisting of 6-monthly evaluations over 12 months, including clinical and cognitive assessments and brain imaging. The Mini-Mental State Examination (MMSE) was used to assess global cognitive level; and the Clinical Dementia Rating (CDR) scale was used to describe disease severity. The education history, quantified as the length in years, was also collected from the subjects.

**Dataset 2** was collected as part of a local research project.^58,61^ The dataset comprises 18 patients with bvFTD, 17 patients with AD, and 24 age-matched controls. Results from AD patients has been previously published.^41^ All the patients in this dataset had been examined by experienced neurologists specialized in memory disorders at the outpatient memory clinic of the Department of Neurology at Oulu University Hospital in Finland. All the patients underwent a series of examinations including neurological examination, a comprehensive neuropsychological evaluation, routine screening laboratory tests, and brain imaging using MRI. MMSE was used to assess their global cognitive level for this study. The modified Frontal Behavior Inventory (FBI) was collected for bvFTD patients. When appropriate, cerebrospinal fluid analyses of β-amyloid_42_, phosphorylated tau, and tau protein or fluorodeoxyglucose positron emission tomography (FDG-PET) were performed to confirm the diagnosis according to clinical practice. Patients were allowed to continue their ongoing medications. Throughout the study, all scans were performed on the same MRI scanner. The mean clinical follow-up time after the fMRI scan was 25 months (0–63 months). Patients presenting progressive aphasia or signs suggesting amyotrophic lateral sclerosis were excluded. The control subjects were interviewed, and MMSE and Beck’s Depression Inventory (BDI) were performed to exclude memory deficits or depression. Any psychiatric or neurological disorders or medications affecting the central nervous system were exclusion criteria for the control group. The inclusion of control subjects required a structural brain MRI free of lesions or significant white matter changes screened by an experienced clinical neuroradiologist.

**Dataset 3** comprised the members of the Northern Finland 1966 Birth Cohort (NFBC1966, http://kelo.oulu.fi/NFBC/index.html). The NFBC1966 is an unselected population birth cohort determined during mid-pregnancy. The cohort is based upon 12,058 children with an expected date of birth during 1966. The live births in this study represent 96% of all births in the region. NFBC1966 members with a possible psychosis were identified.^62,63^ Participants answered questionnaires and underwent psychiatric interviews, cognitive testing, and a brain MRI scan. All participants gave written informed consent and were interviewed using a Structured Clinical Interview for DSM-IV (SCID-I).^59^ Clinical symptoms in participants with schizophrenia were examined using the Positive and Negative Syndrome Scale (PANSS).^64^ The Social and Occupational Functioning Assessment Scale (SOFAS) was used as a rating scale for overall functional level. 23 subjects diagnosed as having SZ and with a technically successful MRI scan formed the patient group of the present study. In the present study, 25 non-psychotic subjects were randomly chosen for the control group. Because both groups were selected from the same birth cohort, the subjects were already age-matched.

### Image acquisition

Each subject was imaged using both functional and structural MRI. Details of the parameters for the MRI scans are shown in Table 2. For additional information on the MRI quality control, see.^58^

**Table 2.** Imaging parameters.

	Dataset 1		Datasets 2 and 3	
	Functional data	Structural data	Functional data	Structural data
Scanner	Siemens Trio Tim		GE Signa HDx	
Field strength (T)	3		1.5	
Sequence	EPI	MP-RAGE	EPI	3DFSPGR BRAVO
TR (ms)/TE (ms)	2000/27	23/2.98	1800/40	12.1/5.2
Duration (number of volumes/time)	240/8 min	NC	202/6 min 4 s	NC
FA (deg)	80	9	90	20
Voxel size (mm)	2.5 × 2.5 × 3.6	1 × 1 × 1	3 × 3 × 3	1 × 1 × 1
Slice thickness (mm)	3	1	4	1

TR: repetition time; TE: echo time; FA: flip angle.

### Neuroimaging processing

Preprocessing followed prior validated approaches, and identical preprocessing and quality control procedures were performed across all subjects, independent of the diagnosis. Preprocessing for all three datasets was conducted using the Oxford Centre for Functional MRI of the Brain Software Library 5.0 (FSL 5.0.11, http://www.fmrib.ox.ac.uk/fsl), exactly as described in.^41^ For fMRI data, this included head motion correction, brain extraction, spatial smoothing, and high-pass temporal filtering.^65–67^ Multi-resolution affine co-registration within the FSL FLIRT software was used to co-register the mean, non-smoothed fMRI, and structural maps of corresponding subjects, and to co-register these imaging data to the Montreal Neurological Institute’s (MNI152) standard space template. Motion analysis: From head motion correction parameters (MCFLIRT), we extracted subject-wise absolute displacement vectors (in mm), which describe the amount of movement in all directions over the entire scan as a marker of gross head motion. Also, relative displacement vectors were extracted as a marker of motion between each fMRI volume. Both vectors were also averaged across volumes to get the mean values. Structural data and gray matter atrophy (GM) maps: after the structural data underwent visual inspection by an experienced neuroradiologist, they were analyzed using FSL-VBM, an optimized voxel-based morphometry analysis (VBM) protocol executed with FSL tools.^66,68–70^ First, structural images were brain-extracted and grey matter-segmented before being registered to the MNI 152 standard space using non-linear registration. The resulting images were averaged and flipped along the x-axis to create a left-right symmetric, study-specific grey matter template. Second, all native grey matter images were non-linearly registered to this study-specific template and “modulated” to correct for local expansion (or contraction) due to the non-linear component of the spatial transformation. The modulated grey matter images were then smoothed with an isotropic Gaussian kernel with a sigma of 3 mm. Finally, voxelwise GLM was applied using permutation-based non-parametric testing, correcting for multiple comparisons across space. For each subject, a total gray matter volume was calculated from the created gray matter maps.

#### Relative brain signal variability maps (CV_BOLD_)

**Figure 1.**
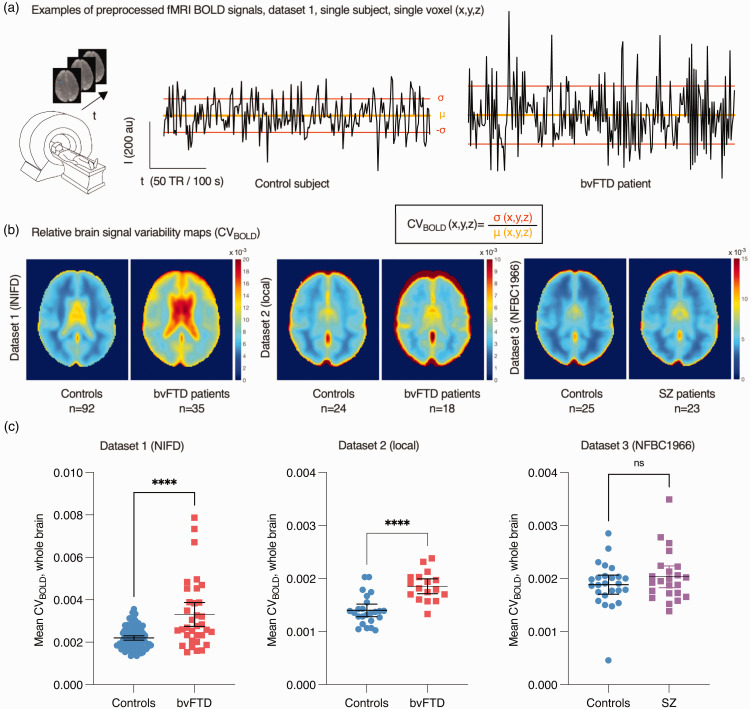
(a) Examples of random single voxel BOLD signal time series and formation of relative brain signal variability maps (CV_BOLD_) based on each voxel’s time series standard deviation (σ) and mean (μ). (b) Group mean CV_BOLD_ maps on axial view and (c) whole-brain average CV_BOLD_ values (mean ± 95% confidence interval). Note that CV_BOLD_ values are dependent on imaging parameters. t-test values: ****p < 0.0001; ns not significant.

We used the temporal coefficient of variation (CV) of the BOLD signal (CV_BOLD_) to measure brain signal variability, where a higher CV value equals greater variability of amplitude of the BOLD signal.^38–41^ CV is also known as relative standard deviation. For each participant, a map of CV_BOLD_ was computed as the relative standard deviation of the BOLD timeseries at each voxel from preprocessed fMRI data:

$${\text{CV}}_{\text{BOLD}}=\text{σ}\left({\text{X}}_{\text{BOLD}}\right)/\text{μ}\left({\text{X}}_{\text{BOLD}}\right)$$

where X_BOLD_ is the voxel time series, σ is the standard deviation and μ is the mean. Representative examples of preprocessed BOLD signals, their standard deviation (SD), mean and the calculated group average CV_BOLD_ maps are shown in Figure 1(b) for all three datasets.

### Statistical analyses

**Figure 2.**
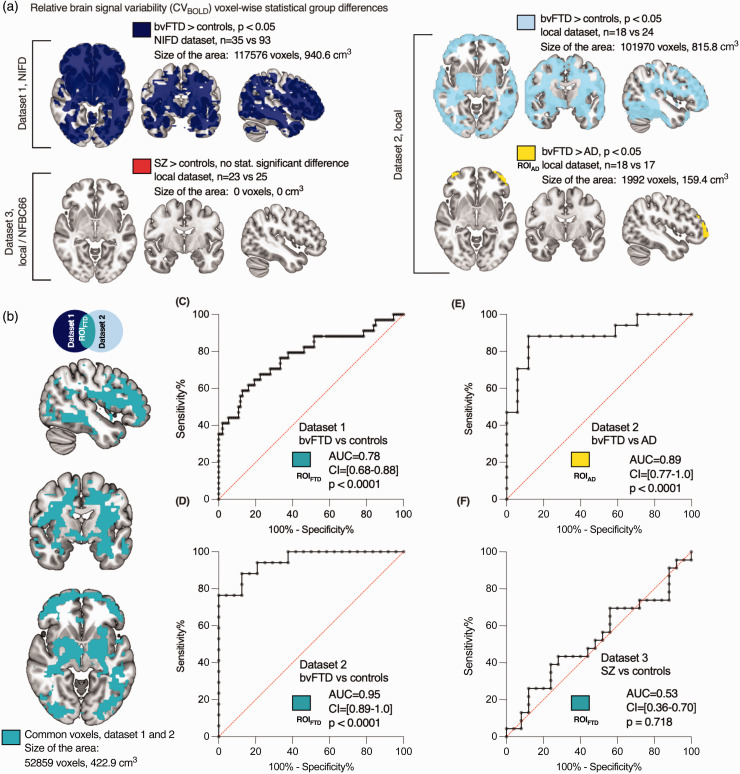
Relative brain signal variability (CV_BOLD_) in patients compared with controls. Differences in CV_BOLD_ were examined using whole-brain voxel-wise analyses. (a) The maps depict group-level differences where CV_BOLD_ is higher in bvFTD patients in both datasets 1 and 2, and where bvFTD patients exhibit higher CV_BOLD_ levels in the frontal areas than AD patients (p < 0.05, family-wise error corrected). Importantly, there were no statistically significant differences observed between schizophrenia patients and controls. (b) Common voxels between datasets 1 and 2, used as the region-of-interest (ROI_FTD_) for further analysis. The sizes of these regions/ROIs were reported in both voxels and cubic centimeters. The 3 D map is available in NIFTI format as a supplementary file. (c–f) Area under the ROC curves (AUC) values were calculated. (c,d) AUC for bvFTD and controls using the ROI_FTD_. (e) AUC for bvFTD vs. AD and (f) AUC for SZ vs. controls. Higher AUC values indicate better discrimination. The 95% confidence intervals (CI) are presented in brackets.

**Figure 3.**
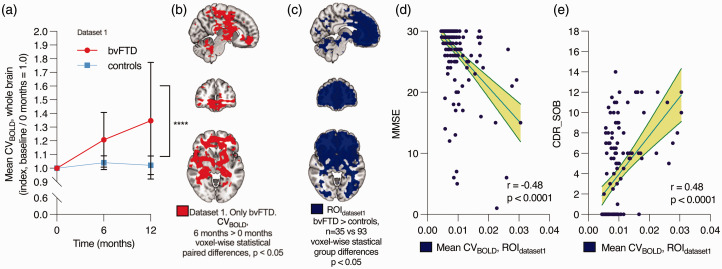
Accuracy, repeatability, and correlation with clinical parameters in dataset 1. (a) Within-individual changes in the average whole-brain CV_BOLD_ over time after baseline imaging (0 months) in bvFTD patients and controls. The data represents the mean ± 95% confidence intervals. Mixed-effects analysis: **** p < 0.0001. (b) Voxel-wise statistically significant difference where CV_BOLD_ increased over 6 months (paired t-test, TFCE-corrected). (c) Voxels displaying group-level differences where CV_BOLD_ is higher in bvFTD patients at baseline (0 months) (same as in Figure 2(a)). This area was used as the region of interest (ROI_dataset1_) in (d–e). (d) Negative correlation between CV_BOLD_ values in this area and Mini-Mental State Examination (MMSE). MMSE scores range from 0 to 30, with a score of 10 to 26 indicating moderate-to-mild cognitive impairment and (e) correlation between CV_BOLD_ in this area and Clinical Dementia Rating (CDR). CDR Sum of boxes (CDR_SOB) scores range from 0 to 18, with a score above 0.5 indicating cognitive impairment, 4.5–9.0 mild dementia, 9.5–15.5 moderate dementia, and 16–18 severe dementia.^95^

All statistical analyses were performed using the GraphPad Prism 9.5.1, GraphPad Software, San Diego, California, USA (www.graphpad.com), unless otherwise stated. Patients were compared to controls in the group-level analysis within each dataset. All results were examined at a p < 0.05 significance level, unless otherwise stated. The χ^2^ test was used to calculate p-values for categorical variables, and the t-test for continuous variables. Voxel-level statistical analysis of imaging data: To examine differences in patients versus controls, between-group contrast comparisons of the various parametric maps were statistically tested using permutation-based nonparametric testing incorporating threshold-free cluster enhancement (TFCE) and correction for multiple comparisons implemented in the FSL randomise tool with 10,000 random permutations. In functional data analysis (CV_BOLD_), relative motion parameters were used as regressors, as in our previous study.^41^ Region-of-interests (ROIs): Statistically significant differences between groups in the voxel-level analysis were also used to define region-of-interest (ROI) segments for some of the further analysis. ROI_AD_ is defined as the set of voxels with statistically significantly increased CV_BOLD_ values in the AD group from dataset 2 and ROI_FTD_ is defined as the set of common voxels with statistically significantly increased CV_BOLD_ values in both dataset 1 and 2. ROI_dataset1_ is defined as voxels where there are group-level differences where CV_BOLD_ is higher in bvFTD patients in dataset 1 in baseline imaging. The size of the ROIs in voxels and volume was also reported. The receiver operating characteristic (ROC) curve and the area under the ROC curve (AUC) were calculated to estimate the feasibility of using CV_BOLD_ as a potential biomarker for bvFTD and AD. We plotted ROC curves to evaluate whether CV_BOLD_ could separate healthy controls from patients in datasets 1 and 2. The mean CV_BOLD_ for each subject was calculated using ROI_FTD_ and ROI_AD_ (Figure 2), and AUC was calculated as a measure of classification accuracy. The bootstrap approach was used to estimate the 95% confidence interval of AUC. Follow-up data in dataset 1 were used to estimate the repeatability and the effect of the bvFTD disease progression on CV_BOLD_. We calculated average CV_BOLD_ within the brain for each subject in dataset 1, and plotted this as a function of time (Figure 3). We analyzed the follow-up CV_BOLD_ data by fitting a mixed model as implemented in GraphPad Prism (Figure 3(a)). This mixed model uses a compound symmetry covariance matrix, and is fit using Restricted Maximum Likelihood (REML). In the absence of missing values, this method gives the same P values and multiple comparisons tests as repeated measures ANOVA. In the presence of missing values (missing completely at random as in here), the results can be interpreted like repeated measures ANOVA. The Pearson correlation coefficient was used as a statistical measure of the strength of a linear relationship between two variables (mean CV_BOLD_ and MMSE/CDR; mean CV_BOLD_ and gray matter volume; mean CV_BOLD_ and education history). Visualization: Most of the data were plotted using GraphPad Prism 9. fMRI data were plotted using Matlab (Figure 1) or MRIcroGL (Figures 2 and 3, https://www.mccauslandcenter.sc.edu/mricrogl/).

No power analysis was performed before the study. Nevertheless, the dataset sizes were in line with previous experiments using functional imaging in patients with a neurodegenerative disease. Data collection and further analyses were not performed blind to the conditions of the experiment. Preprocessing and analysis of neuroimaging data included standard automated analytic FSL pipelines, which were agnostic to the diagnostic and demographic characteristics of the data.

## Results

### Relative brain signal variability is increased in bvFTD but not in SZ

Physiological relative brain signal variability was estimated using CV_BOLD_ (Figure 1). Compared to healthy controls scanned using the same scanner, the average CV_BOLD_ calculated from whole brain was significantly increased in bvFTD in both datasets 1 (mean 3.3 × 10^−3^ vs. 2.2 × 10^−3^, p < 0.0001, t = 5.891, df = 12) and 2 (mean 1.9 × 10^−3^ vs. 1.4 × 10^−3^, p < 0.0001, t = 5.150, df = 39) (Figure 1(c)). There was no statistically significant difference between SZ and controls (mean 2.0 × 10^−3^ vs. 1.9 × 10^−3^, p = 0.26, t = 1.135, df = 46) (dataset 3, Figure 1(c)).

### Patterns of increased relative brain signal variability in bvFTD and AD

To investigate the anatomical distribution of the altered brain signal variability, we did whole-brain voxel-wise analysis, where bvFTD patients showed an increased CV_BOLD_ (p < 0.05, family-wise error corrected for multiple comparisons, and with head-motion parameter used as a regressor). This analysis revealed widespread clusters of voxels distributed around the basal ganglia, periventricular white matter, and frontal and occipital cortices in bvFTD patients in datasets 1 and 2 (Figure 2). Most parts of the frontal cortical grey matter, lateral temporal areas, and periventricular white matter were shown to have a significantly increased CV_BOLD_ (Figure 2(b)). Symmetrically, both the putamina (except one third of the frontal tips), capsula externa, lateral thalami, and amygdalae and medial caudate nuclei were also involved in both datasets. Two thirds of both capsula interna were symmetrically spared in the middle of large changes in both datasets. The cerebellum was also involved symmetrically behind the IV ventricle.

Results from the AD patients have been published previously, where we showed increased brain signal variability in AD compared to controls.^41^ In this study we compared CV_BOLD_ maps of bvFTD and AD patients: bvFTD patients showed increased CV_BOLD_ symmetric bilateral frontopolar cortices compared to AD patients, Figure 2(a).

Again, in the more spatially detailed analysis there were still no statistically significant differences between SZ and controls. Furthermore, there were also no regions with significantly higher CV_BOLD_ in controls compared to bvFTD or SZ patients.

### Replicability of increase in brain signal variability

To assess the potential for differential diagnosis of the diseases using CV_BOLD_, we assessed the anatomical concordance of the regions of increased CV_BOLD_ in the two independent bvFTD datasets in this study (Figure 2(b)). The anatomical patterns of increased CV_BOLD_ were markedly alike in both datasets 1 and 2, even though the imaging setups differed (Table 2). There were altogether 52,859 common 2 mm voxels (volume 423 cm^3^) that showed significantly increased CV_BOLD_ in both bvFTD datasets. This 3 D map is available in NIFTI format as a supplementary file. Quantitatively, the spatial cross-correlation of significantly altered voxels was 0.65 measured using FSLCC software, part of FSL (FMRIB's Software Library^70,71^)

### Accuracy of brain signal variability in discriminating between bvFTD patients, controls, and AD patients

To analyze diagnostic accuracy and repeatability of increased brain BOLD signal variability we used receiver operating characteristics (ROC) curves: the area under the curve (AUC) was calculated to estimate the viability of CV_BOLD_ as a biomarker for bvFTD. Both datasets 1 (AUC = 0.78, 95%-CI 0.68–0.88, p < 0.0001, Figure 2(c)) and 2 (AUC = 0.95, 95%–CI 0.89–1.0, p < 0.0001, Figure 2(d)) demonstrated that CV_BOLD_ was a good to excellent discriminator between bvFTD patients and controls. Additionally, CV_BOLD_ clearly separated bvFTD and AD patients (AUC =0.89, 95%–CI 0.77–1.0, p < 0.0001, Figure 2(e)). ROC curve analysis showed that CV_BOLD_ could not be used to discriminate between SZ and controls (Figure 2(f)).

### The increase in brain signal variability is repeatable and associated with lower cognitive scores

We analyzed the repeatability and possible effect of disease progression of these findings using the follow-up data in dataset 1. In the control group, average CV_BOLD_ values within the whole brain were relatively stable over a 12-month period (+2% on average) (Figure 3(a)), thus indicating good repeatability. Over the follow-up period, the CV_BOLD_ increased only in the bvFTD group (+35% on average). The repeated measures were analyzed using the mixed effects model. The difference between bvFTD and controls was statistically significant, F(2, 106) = 12.91, p < 0.0001. The predicted mean of bvFTD was 1.180 and controls 1.021. In a pairwise voxel-based analysis, there was an increase in CV_BOLD_ in the periventricular and frontal areas over 6 months (Figure 3(b)) and 12 months (not shown, similar to Figure 3(b)).

MMSE, global CDR, and CDR Sum of Boxes (CDR SOB) scales were used to test for cognitive impairment in the bvFTD participants (Table 1). There was a negative correlation between MMSE scores and average CV_BOLD_ (r = −0.48, p < 0.0001) and positive correlation between CDR SOB scores and average CV_BOLD_ (r = 0.48, p < 0.0001), meaning that increased BOLD signal variability was associated with lower cognitive function scores (Figure 3(d) and (e)). There was also a positive correlation with global CDR scores (r = 0.46, p < 0.0001).

### The impact of gray matter atrophy, head motion, sex, and education on CV_BOLD_ increase

**Figure 4.**
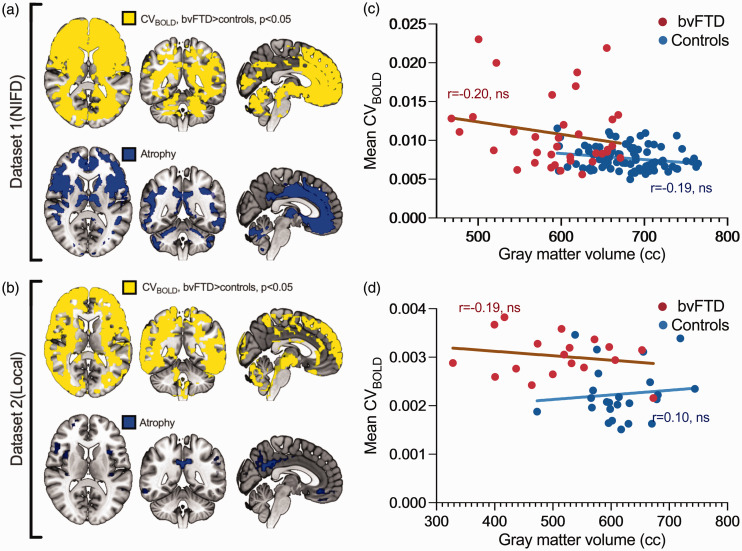
Group-level differences in CV_BOLD_ and voxel-based morphometry results. a-b shows the area where CV_BOLD_ is statistically significantly higher in bvFTD patients compared to controls (marked with yellow). Correspondingly, gray matter volume is lower in bvFTD patients compared to controls, indicated in blue. Panels a and b present p-value maps (family-wise error corrected, p < 0.05). Panels c and d depict the correlation between gray matter volume and whole-brain mean CV_BOLD_ values.

The effect of GM atrophy on CV_BOLD_ values was analyzed (Figure 4) in bvFTD datasets. Anatomically, the increase in CV_BOLD_ in bvFTD was more widespread than atrophy (Figure 4(a) and (b)). There was no statistically significant correlation between the mean CV_BOLD_ values and the volume of GM (Figure 4(c) and (d)). There was small, but statistically significant difference in the relative motion between SZ patients and their controls (0.08 vs 0.06), but not between bvFTD patients and their controls (Supplementary Table 1). In further analysis, relative motion parameters were used as regressors, as in our previous study.^41^ In datasets 2 and 3, there were no statistically significant differences between the sexes of the participants. In dataset 1, the use of indicated sex as a regressor in voxel-level analysis using FSL randomise produced statistical maps that were 99% identical to those without a regressor, with an fslcc spatial correlation coefficient r = 0.99 (Supplementary Figure 1). Educational data was available only for dataset 1, where there were no statistically significant differences between bvFTD patients and controls (18 vs. 21 years). The correlation between the mean CV_BOLD_ and education history was found to be weak (r = −0.12, not statistically significant, 95% CI −0.24 to 0.00).

## Discussion

In this study, we investigated the relative brain signal variability of the BOLD signal (CV_BOLD_), measured using fMRI, in two different disorders: the behavioral variant of frontotemporal dementia (bvFTD) and schizophrenia (SZ). We also compared the results with our previous findings of increased CV_BOLD_ in Alzheimer’s disease (AD).^41^ We found that CV_BOLD_ was increased in bvFTD patients, but not in SZ patients. We identified increased CV_BOLD_ in an international longitudinal bvFTD dataset, and replicated these findings in an independent local dataset. The follow-up data from the longitudinal FTLDNI study showed that the CV_BOLD_ measure is relatively stable in controls but increases with time in individual bvFTD patients scanned six months and one-year later: this indicates an association between elevated CV_BOLD_ and disease progression. Furthermore, we found that the increase in CV_BOLD_ was associated with lower cognitive function scores (MMSE and CDR). Additionally, we found that CV_BOLD_ could accurately discriminate bvFTD patients from controls (ROC AUC = 0.78–0.95, p < 0.0001) and bvFTD patients from AD patients (ROC AUC = 0.89, p < 0.0001). The effect of brain atrophy on CV_BOLD_ was investigated in this study as it may explain some of the observed results. However, the changes in CV_BOLD_ are more widely localized than atrophy, and there was no correlation between mean CV_BOLD_ values and GM volume. Given the extent of this change, the contribution of the partial volume effect is also presumed to be small. Atrophy GM atrophy patterns in bvFTD were in line with previous literature^72–75^

Our initial idea to apply the CV to BOLD signals was inspired by laser speckle contrast imaging (LSCI), a wide-field optical imaging technique capable of visualizing blood flow used in imaging vascular structures and their associated hemodynamics.^76–78^ We hypothesized that the CV in fMRI data analysis allows for the quantification of variability in BOLD signals across the whole brain where a higher CV value equals greater variability of amplitude of the BOLD signal, which is closely aligning with its use in LSCI contrast. Increased BOLD signal variability using similar methods has been detected in AD.^79,80^ Makedonov et al. have also shown that the BOLD signal variation reflects intracranial pulsatility effects in elderly patients with small vessel and chronic kidney disease.^81^ A similar study showed a close association of increased intracranial pulsatility (rather than with low global CBF) with the small vessel disease features such as cerebral white matter lesions, cerebral microbleeds, perivascular spaces, brain atrophy, and lacunar infarcts.^82^ Similarly, higher white matter hyperintensity burden has been shown to be associated with greater BOLD signal variability in right temporal regions, and lower scores on a measure of global executive functioning.^83^

To the best of our knowledge, no previous studies have found increased CV_BOLD_ in bvFTD patients, although increased CV_BOLD_ in AD patients has been reported by us and other research groups.^41,79,80^ In this study, not only did we find increased CV_BOLD_ in bvFTD patients, we also determined that the spatial localization of this increase differs between these diseases: this makes it possible to discriminate between bvFTD and AD patients. Our results extend this line of research by demonstrating the potential clinical usefulness of CV_BOLD_ as a biomarker for bvFTD diagnosis and monitoring disease progression.

In AD, ultrafast fMRI has determined that the cardiovascular pulse propagation inside the brain parenchyma is more variable, and that the main driver for increased CV_BOLD_ is intracranial cardiorespiratory pulsation: this is thought to link with glymphatic clearance.^35,37^ The impulse speeds were mostly increased in narrowed peripheral arteries but, importantly, a reversed impulse propagation was detected in (para)hippocampal areas known to present increases in the permeability of the blood brain barrier (BBB) irrespective of β-amyloid/tau depositions in early AD.^29,37,41^

Interestingly, a recent study has suggested that regional glymphatic dysfunction may also contribute to the bvFTD pathogenesis.^84^ Glymphatic function, especially in the anterior and middle regions of brain, was found to be impaired in bvFTD. Moreover, regional glymphatic function showed a spatial correlation with the bvFTD-related metabolic pattern and clinical symptoms. Future studies should investigate the physiological basis of CV_BOLD_ alterations in bvFTD and whether these changes relate to dysfunction of the glymphatic system, especially within the paravascular solute pathways in the BBB.

In addition to investigating physiological pulsations and the glymphatic system, another compelling avenue for future research is exploring the impact of our findings on aspects such as the hemodynamic response function (HRF)^85,86^ and functional connectivity. Variability of HRF (HRFv) could alter resting-state functional connectivity findings.^87,88^ However, most algorithms for HRF estimation are tailored for task-related fMRI data, and only a few are applicable to resting-state protocols. Furthermore, identifying the timing information of spontaneous events for HRF estimation is challenging without concurrent electrophysiological recordings.^89^ It would be interesting in the future to explore the relationship between our results and such algorithms, especially by utilizing multimodal ultrafast fMRI sequences.

In contrast to bvFTD, we did not find any significant differences in brain signal variability between SZ patients and controls. This finding is somewhat unexpected, as a previous study showed increased variance of BOLD signal in SZ.^90^ On the other hand, variance calculated as (standard deviation)^2^ is not the same as coefficient of variation which takes also account the average intensity of the volumes (standard deviation/mean).^91^ In our previous study, familial risk for psychosis or genetic risk for SZ did not appear to be related to CV_BOLD_ in the brain.^92^ Further research using more advanced techniques such as fast MRI may be needed to fully understand the relationship between brain signal variability and SZ.

Our results demonstrate that CV_BOLD_ has an excellent accuracy and repeatability in discriminating bvFTD patients from controls and AD patients. This finding suggests that CV_BOLD_ may be a useful diagnostic tool for distinguishing bvFTD from other neurodegenerative disorders that share similar clinical symptoms, such as AD. Additionally, the high repeatability of brain signal variability suggests that it may be a reliable marker for monitoring disease progression and treatment response in bvFTD patients.

Finally, we found that the increase in brain signal variability was associated with more severe clinical cognitive impairment scores (MMSE and CDR) in bvFTD patients. This finding suggests that increased brain signal variability may be a useful biomarker for tracking disease severity in bvFTD patients. However, further larger prospective longitudinal studies are needed to confirm this association and to determine the potential clinical usefulness of brain signal variability as a prognostic marker for bvFTD.

Our work features some limitations. First, AD and bvFTD diagnoses were based on clinical expertise but with no pathological confirmation. However, the diagnostic criteria for both AD and bvFTD fulfilled standard diagnostic guidelines. Secondly, there is a certain lack of standards in the existing literature with respect to methodology and terminology in brain signal variability research. Also, a common issue in fMRI scanning has been the dependence of the results on scanner and imaging parameters. However, the concordance of the present CV_BOLD_ results in bvFTD from two independent datasets seems to indicate a consistent pattern of bvFTD-related changes, despite quite substantial differences in the different vendor scanners and scanning parameters. As people age, structural and functional changes in the brain occur that can alter the variability and complexity of brain signals. Previous studies have shown that variability in spontaneous brain activity can decrease with age.^93,94^ In this study we used age-matched controls to mitigate any potential confounding effects arising from the established influence of age on brain signal variability. One of the strengths of this study is the inclusion of the white matter signal in the analyses. However, a limitation is that we did not analyze the effect of white brain matter atrophy on the results to the same extent as the gray matter atrophy. Prospective studies of larger replication samples with longitudinal fMRI should serve to establish the diagnostic usefulness of the present methods.

## In conclusion

Our study demonstrates that brain signal variability is increased in bvFTD but not in SZ patients, and that it has excellent accuracy and repeatability in discriminating bvFTD patients from both controls and AD patients. Furthermore, our results suggest that brain signal variability may be a useful biomarker for monitoring disease progression and treatment response in bvFTD patients. However, further research is needed to fully understand the potential clinical applications of this technique.

## Supplemental Material

sj-zip-1-jcb-10.1177_0271678X241262583 - Supplemental material for The relative brain signal variability increases in the behavioral variant of frontotemporal dementia and Alzheimer’s disease but not in schizophrenia Supplemental material, sj-zip-1-jcb-10.1177_0271678X241262583 for The relative brain signal variability increases in the behavioral variant of frontotemporal dementia and Alzheimer’s disease but not in schizophrenia by Timo Tuovinen, Jani Häkli, Riikka Rytty, Johanna Krüger, Vesa Korhonen, Matti Järvelä, Heta Helakari, Janne Kananen, Juha Nikkinen, Juha Veijola, Anne M Remes, Vesa Kiviniemi and on behalf of the Frontotemporal Lobar Degeneration Neuroimaging Initiative in Journal of Cerebral Blood Flow & Metabolism

## Data and code availability statement

The individual data from this study cannot be shared because of privacy issues of clinical data. Data from the datasets are available for research only after ethical approval for a specific project. NIFD dataset is available from its own online repositories [Neuroimaging In Frontotemporal Dementia (NIFD/LONI)]. The code for the data analysis of this study is available from the corresponding author on reasonable request.
